# Efficacy of electroacupuncture therapy in patients with functional anorectal pain: study protocol for a multicenter randomized controlled trial

**DOI:** 10.1007/s00384-024-04628-5

**Published:** 2024-04-22

**Authors:** Yahong Xue, Huifen Zhou, Yusha Zeng, Chen Wang, Yun Yang, Xiaopeng Wang, Zongqi He, Yibo Yao, Xiaofeng Wang, Zhimin Fan

**Affiliations:** 1 Department of Anorectal, Nanjing Hospital of Chinese Medicine Affiliated to Nanjing University of Chinese Medicine, No.157 Daming Road, Qinhuai District, Nanjing, 210022 People’s Republic of China; 2 Graduate School of Nanjing University of Chinese Medicine, Nanjing, 210023 People’s Republic of China; 3 Department of Anorectal, Longhua Hospital of Shanghai University of Traditional Chinese Medicine, Shanghai, 200032 People’s Republic of China; 4 Anorectal Department, Yinchuan Traditional Chinese Medicine Hospital, Yinchuan, 750010 People’s Republic of China; 5 Department of Anorectal, Suzhou Hospital of Traditional Chinese Medicine Affiliated to Nanjing University of Chinese Medicine, Suzhou, 215009 People’s Republic of China

**Keywords:** Functional anorectal pain, Electroacupuncture, Baliao acupoint, Study protocol, Randomized controlled trial

## Abstract

**Background:**

Some Chinese scholars have initially explored the efficacy of electroacupuncture at Baliao acupoint in patients with functional anorectal pain (FAP). However, their studies are performed in a single center, or the sample size is small. Therefore, we aim to further explore the efficacy of electroacupuncture at Baliao acupoint on the treatment of FAP.

**Methods:**

In this multicenter randomized controlled trial, 136 eligible FAP patients will be randomly allocated into an electroacupuncture group or sham electroacupuncture group at a 1:1 ratio. This trial will last for 34 weeks, with 2 weeks of baseline, 4 weeks and 8 weeks of treatment, and 1, 3, and 6 months of follow-up. Outcome assessors and statisticians will be blind. The primary outcome will be clinical treatment efficacy, and secondary outcomes will be pain days per month, quality of life, psychological state assessment, anorectal manometry, pelvic floor electromyography, and patient satisfaction.

**Discussion:**

Results of this trial will be contributed to further clarify the value of electroacupuncture at Baliao acupoint as a treatment for FAP in the clinic.

**Trial registration:**

This trial has been registered in Chinese Clinical Trial Registry https://www.chictr.org.cn/ (ChiCTR2300069757) on March 24, 2023.

**Supplementary Information:**

The online version contains supplementary material available at 10.1007/s00384-024-04628-5.

## Introduction

Functional anorectal pain (FAP) is an unexplained and nonorganic pain [1]. Rome IV categorized FAP into three types: proctalgia fugax, levator ani syndrome, and unspecified functional anorectal pain [2]. The latter two types are distinguished by whether there is traction pain on the puborectalis [3]. The prevalence of anorectal pain caused by all reasons or levator ani syndrome is 11.6% and 6.6%, respectively [3]. FAP patients often have mental and emotional disorders, which seriously affect their mental health and quality of life [4].

The pathophysiology of FAP remains unclear. Excessive contraction and high tension of pelvic floor muscle have been widely believed as one of the possible mechanisms [4]. A study has indicated that levator ani syndrome was closely associated with the pelvic floor spasm [5]. In addition, paroxysmal anal hypermobility was reported as an important feature of proctalgia fugax [6]. At present, a range of interventions have been reported for FAP, such as medications, biofeedback, electrogalvanic stimulation (EGS), botulinum toxin injection, and sacral nerve modulation (SNM) [1, 3]. A recent meta-analysis indicated that acupuncture should be considered a new and alternative intervention in future studies [7].

Acupuncture at Baliao acupoint has been reported to treat pelvic floor dysfunctions [8, 9]. Shen et al. have found that the level of anal pain and incidence of urinary retention were lower after the electroacupuncture at Baliao acupoint than the control in patients undergoing stapled hemorrhoidopexy [8]. Liu et al. have reported that electroacupuncture at Baliao acupoint significantly improved bladder capacity and compliance in patients with detrusor overactivity after stroke and reduced detrusor leak point pressure [9]. In China, some scholars have initially investigated the effect of electroacupuncture at Baliao acupoint on FAP [10, 11]. It had been reported that electroacupuncture at Baliao acupoint was superior to biofeedback in efficacy, visual analogue scale (VAS) score, and quality of life [10]. The study of Yu et al. showed that electroacupuncture at Baliao acupoint combined with biofeedback significantly promoted the clinical efficacy and decreased the VAS score compared to biofeedback alone [11]. Because of the small sample size or the single center of these studies [10, 11], the efficacy of electroacupuncture at Baliao acupoint in the treatment of FAP needs to further explore.

Considering this, this study aims to further explore the effect of electroacupuncture at Baliao acupoint on the treatment of FAP through a larger sample size, multicenter, randomized controlled trial.

## Methods

### Study design

**Fig. 1:**
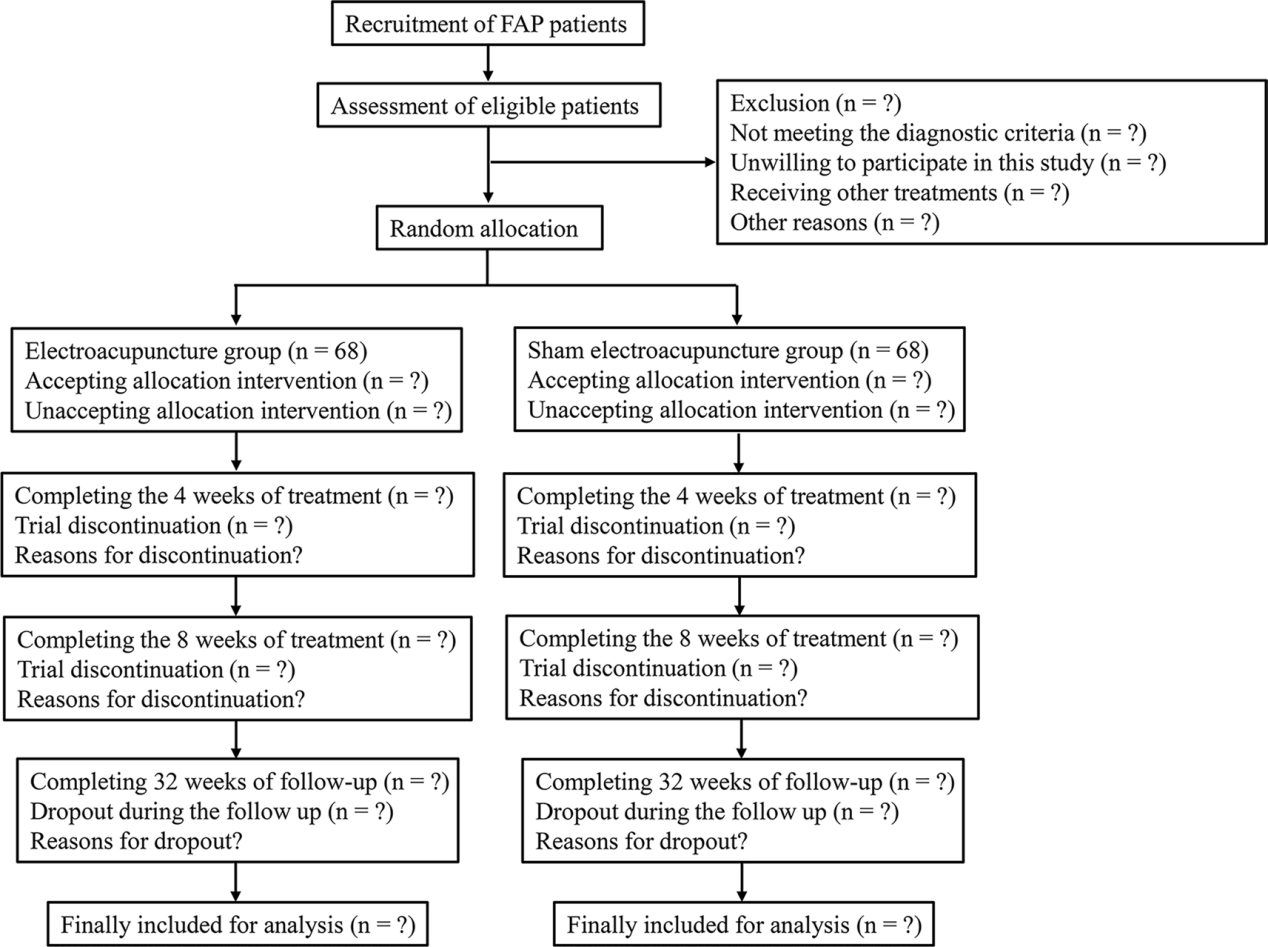
Flowchart showing the selection process of FAP patients

This is a multicenter, paralleled, randomized, controlled trial. The eligible patients will be randomly divided into the treatment group (electroacupuncture group) and control group (sham electroacupuncture group) at a 1:1 ratio. This study will last for 34 weeks, including 2 weeks before randomization (baseline), 8 weeks of treatment, and 24 weeks of follow-up. Follow-up will be conducted at baseline, 4 weeks of treatment, 8 weeks of treatment, and 1 month (12 weeks), 3 months (20 weeks), and 6 months (32 weeks) after the treatments. This study protocol conforms to the Consolidated Standards of Reporting Trials (CONSORT) [12] and Standards for Reporting Interventions in Clinical Trials for Acupuncture (STRICTA) [13]. This study followed the Declaration of Helsinki and has been approved by the Institutional Review Board of Nanjing Hospital of Chinese Medicine Affiliated to Nanjing University of Chinese Medicine, Longhua Hospital of Shanghai University of Traditional Chinese Medicine, Yinchuan Traditional Chinese Medicine Hospital, and Suzhou Hospital of Traditional Chinese Medicine Affiliated to Nanjing University of Chinese Medicine, approval no. KY2023012. Written consent form will be obtained from all patients. This study has been registered in the Chinese Clinical Trial Registry (registration no. ChiCTR2300069757) on March 24, 2023. The details of study design are demonstrated in Fig. 1, and the trial schedule is presented in the Supplementary information Table 1.

### Participants

Patients will be recruited from the outpatient clinic and inpatients in the Anorectal Department of Nanjing Hospital of Chinese Medicine Affiliated to Nanjing University of Chinese Medicine, Longhua Hospital of Shanghai University of Traditional Chinese Medicine, Yinchuan Traditional Chinese Medicine Hospital, and Suzhou Hospital of Traditional Chinese Medicine Affiliated to Nanjing University of Chinese Medicine. The recruitment strategies include putting up posters and distributing information on the WeChat Public Platform (the largest social media platform in China). All patients will be informed of the details of the study (objective, procedure, and potential risks) in written form, and the informed consent will be signed before the allocation.

### Inclusion criteria

Patients meeting the following criteria will be included: (1) meeting the diagnostic criteria of levator ani syndrome and unspecified functional anorectal pain (Rome IV criteria), (2) with chronic or recurrent FAP (pain lasting for at least 30 min each time, occurring at least once a week, and presenting ≥ 6 months), (3) aged 20–70 years old, (4) not receiving acupuncture treatment within 3 months before the enrollment, and (5) voluntarily participating in this study and signing the informed consent form.

Levator ani syndrome is diagnosed as the following criteria: chronic or recurrent rectal pain, lasting for at least 30 min, traction pain on puborectalis, excluding other causes of rectal pain, such as inflammatory bowel disease, intramuscular abscess or anal fissure, thrombotic hemorrhoids, and prostatitis. Unspecified functional anorectal pain is diagnosed as the following criteria: meeting the diagnostic criteria for levator ani syndrome but not presenting traction pain on puborectalis.

### Exclusion criteria

Patients will be excluded if they are (1) with pain caused by organic diseases, such as anal fissure, perianal abscess, and hemorrhoids; (2) with a history of anal and pelvic floor surgery within the past year; (3) with a history of pelvic and spinal cord injuries; (4) with severe cardiovascular, cerebrovascular, liver, kidney, respiratory, and hematopoietic diseases or malignant tumors; (5) taking anti-anxiety or anti-depression drugs to control illness (for the past 3 months), or currently combining with mental illness (confirmed); and (6) pregnant, lactating, and menstrual women.

### Elimination criteria

Patients will be eliminated if they are (1) mistakenly included patients who do not meet the inclusion criteria; (2) patients who had not been treated according to the protocol specified in this study, and with incomplete basic information and condition information, which affects the evaluation of clinical efficacy and safety; (3) patients with poor compliance and voluntarily withdrawing during the treatment process; and (4) patients using the prohibited treatment methods in this study protocol or changing the treatment methods themselves during the treatment process.

After the subject is eliminated, the supervising doctor will contact the subject by phone calls or letters to inquire about the reasons, record the time of the last treatment, and complete the assessment items that can be completed. For subjects withdrawn from the trial due to adverse reactions or ineffective treatment, the supervising doctor will take corresponding measures based on the actual situation. Intention-to-treat analysis will be conducted on all subjects who are eliminated or lost to follow-up after the end of the trial.

### Criteria for discontinuing the trial

The trial will be discontinued if the following conditions occur: (1) patients exhibit serious adverse reactions during the study and are not suitable to continue the study; (2) patients require emergency measures for the reason that serious complications are presented or the condition is worsened during the study; (3) patients withdraw from the clinical study midway; and (4) patients do not cooperate and obey the treatment, which is not changed after repeated explanations from clinical doctors. The reasons and time for the discontinuation and withdrawal should be recorded by researchers.

### Randomization and blinding

The stratified block randomization will be taken in this study. Randomization will be stratified according to the center, and the block size is 4. Randomization is generated by SAS software (SAS Institute Inc., Cary, NC, USA), and allocation will be hidden through opaque sealed envelopes. The treatment allocation corresponding to serial no. 001–136 is listed (random coding table), and the serial number is corresponded to the number of subjects. A designated person will keep the random coding table. After subjects are selected, researchers will inform the random coding table keeper of the corresponding number of the subject. The keeper will give instructions to the subject to enter which group according to a random coding table. The researchers will record the instructions and comply with the corresponding allocation according to the instructions.

Due to the particularity of acupuncture operation, it is difficult to make operators and patients be blind. Therefore, outcome assessors and statisticians will be blind to reduce the risk of bias.

## Intervention

Patients will be given 24 times of treatments during 8 consecutive weeks, with each treatment lasting 30 min and 3 times of treatments per week (once every 2 days at an ideal condition). All the acupuncture therapists in this study have obtained the certificate of licensed practicing physician and have more than 5 years of experience in acupuncture operation. In order to improve the consistency of treatment in each center, the acupuncture therapists will receive a standard training before the treatment.

### Electroacupuncture group

Patients will be placed in a prone position, and local skin is routinely disinfected. The bilateral Zhongliao (BL33) and bilateral Xialiao (BL34) are selected. BL34 is located in the sacral region and faces the fourth posterior sacral foramina. For BL34, a 0.35 × 0.75-mm needle (Jiajian Medical Equipment Co., Ltd.) is vertically inserted into the fourth posterior sacral foramina. BL33 is located in the sacral region and faces the third posterior sacral foramina. For BL33, a 0.35 × 0.75-mm needle (Jiajian Medical Equipment Co., Ltd., Wuxi, China) is inserted into the third posterior sacral foramina and is at an angle of 70° to the coronal plane of the human body. After inserting, the needle is gently lifted, inserted, twirled, and rotated to achieve a Deqi sensation. After achieving the Deqi sensation, even a reinforcing-reducing method is conducted. The electroacupuncture (HM6805-II, Hengming Medical Company, Chengdu, China) is given in bilateral BL33 and BL34, with a dilatational wave, a frequency of 2/15 Hz, and a stimulation intensity of 1-5 mA, based on the patient’s tolerance level. The needle is left in the patient’s body for 30 min, and patients are not needled during this process.

### Sham electroacupuncture group

The bilateral sham BL33 (opening 20 mm outward at actual BL33) and bilateral sham BL34 (opening 20 mm outward at actual BL34) are selected, with shallow needling (0.35 mm × 25 mm, Jiajian Medical Equipment Co., Ltd., Wuxi, China) of 3–5 mm. The electrode placement and parameter settings of the electroacupuncture are the same as those of the electroacupuncture group, but the electricity is not turned on. After needling, there is no operation to achieve the Deqi sensation.

### Adverse event

The adverse reactions such as needle fainting, needle sticking, needle bending, and needle breaking during the electroacupuncture treatment, as well as adverse events such as empyrosis and getting blisters during the moxibustion, should be recorded. All adverse events should be treated appropriately based on the patient’s condition until the condition is stabilized. The adverse events can be processed by one or more of the following: taking no measures (mainly observation); adjusting electroacupuncture treatment measures such as reducing treatment frequency, reducing stimulation, and temporarily or permanently discontinuing electroacupuncture treatment; and providing non-drug therapies.

### Data collection and management

A case report form (CRF) will be used to collect study-related data. The CRF must be completed for every subject by the researcher. The completed CRF will be reviewed by clinical researchers and supervisors, and then be handed over to the data administrators. Data administrators will develop the data management system according to the CRF. To ensure the accuracy of data, two data administrators will independently enter and proofread the data. For queries in the CRF, data administrators will issue an inquiry to the researchers, and correct, confirm, and enter the data based on the researchers’ answer. After confirming the accuracy of all data, the database will be locked by the main researcher, sponsor, and statistical analyst. The locked data cannot be modified.

## Outcome measurement

### Primary outcome

#### Clinical treatment efficacy

Referring to the Guiding rules of clinical research of new Chinese medicine [14], the VAS score will be used to evaluate efficacy. VAS scores at baseline, 4 weeks and 8 weeks of treatment, and 1, 3, and 6 months of follow-up will be recorded, and clinical treatment efficacy is evaluated using the efficacy index. Efficacy index is calculated as [(pre-treatment VAS score − post-treatment VAS score) ÷ pre-treatment VAS score] × 100%. Curing is defined as the pain symptom completely disappears, and the VAS score reduction rate is ≥ 95%. Apparent effect is defined as the pain symptom broadly disappears, with occasional discomfort, and the VAS score reduction rate is ≥ 70% and < 95%. General effect is defined as the pain symptom is improved, with the non-ignorable discomfort during onset which does not affect life and work, and the VAS score reduction rate is ≥ 30% and < 70%. No effect is defined that the pain symptoms are not apparently improved, with the pain affecting life and work during onset, and the VAS score reduction rate is < 30%.

### Secondary outcomes

#### Pain days per month

The number of pain days per month will be evaluated based on 30 days of pain logs recorded before the baseline period, 4 weeks and 8 weeks of treatment, and 1, 3, and 6 months of follow-up. The pain logs will not be obtained by the therapists.

#### Quality of life

The quality of life will be assessed at the baseline period, 4 weeks and 8 weeks of treatment, and 1, 3, and 6 months of follow-up using a 36-Item Short Form Health Survey questionnaire (SF-36) [15]. SF-36 assesses the quality of life of the subjects based on 8 aspects: physical activities, physiological function, bodily pain, health perceptions, vitality (energy and fatigue), social activities, emotional function, and mental health [15]. The higher score in each aspect indicates the better the quality of life in this aspect [15].

#### Psychological state assessment

Psychological state will be evaluated using a Self-rating Anxiety Scale (SAS) and Self-rating Depression Scale (SDS) [16, 17]. Psychological state will be evaluated at the baseline period, 8 weeks of treatment, and 3 and 6 months of follow-up.

#### Anorectal manometry

Anorectal manometry will be performed to assess the anorectal function (anal sphincter function, rectal sensory function, and anorectal reflex) using the high-resolution anorectal manometry instrument. Outcome measures (1) anal sphincter function: anal resting pressure, maximum anal systolic pressure, rectal defecation pressure, and high-pressure zone; (2) rectal sensory function: initial threshold, defecation sensation threshold, and maximum tolerance; (3) anorectal reflex: defecation relaxation reflex, anorectal contraction reflex, and anorectal inhibition reflex. Anorectal manometry will be evaluated at the baseline period, 8 weeks of treatment, and 6 months of follow-up.

#### Pelvic floor surface electromyography

Pelvic floor electromyography will be performed to assess the pelvic floor muscle function by using a pelvic floor biofeedback therapeutic instrument with the Glazer Protocol. The process and detection indicators of Glazer Protocol are shown as the following: (1) pre-baseline: resting for 1 min, detecting amplitude and coefficient of variation (coefficient of variation = standard deviation/mean, the same below); (2) rapid contractions: conducting five rapid contractions with a 10-s rest between each contraction, detecting contraction response time and maximum contraction amplitude; (3) continuous contractions: conducting five contractions with resting for 10 s and continue to contract for 10 s, detecting the contraction amplitude, coefficient of variation, and median frequency; (4) endurance contraction: contracting continuously for 1 min, detecting amplitude, coefficient of variation, and median frequency; (5) post-baseline: resting for another 1 min, detecting amplitude and coefficient of variation. Pelvic floor electromyography will be evaluated at the baseline period, 8 weeks of treatment, and 6 months of follow-up.

#### Patient satisfaction

Patient satisfaction will be evaluated at the 6 months of follow-up using a 5-point Likert scale [18]. The scores range from 1 to 5 (1 = highly not satisfied, 2 = not satisfied, 3 = neutral, 4 = satisfied, and 5 = highly satisfied) [18].

#### Quality control

To ensure the quality of the study, acupuncturists, research assistants, and statisticians must receive unified training. A multi-center experimental coordination committee will be established to be responsible for the implementation of the entire study and resolving issues related to the study. Experienced personnels will serve as inspectors to guarantee the rights and interests of the subjects, confirm accuracy and completeness of the data, ensure the compliance of study to approved protocols and relevant regulations, and monitor the center regularly.

#### Sample size calculation

The sample size is calculated based on the previous study [19]. This study assumes a 70% effective rate in the electroacupuncture group and a 40% effective rate in the sham electroacupuncture group. *α* is taken as 0.05, and 1- *β* (power) is taken as 0.9. PASS 11.0 software (NCSS, LLC. Kaysville, Utah) is used to calculate the sample size, and 57 subjects will be required for each group. Considering a 15% dropout rate, 67 subjects are required for each group, and a total of 134 subjects are required. Due to the sample size jointly responsible by 4 centers and block randomization, each center will provide 34 subjects, and a total of 136 subjects are required.

### Statistical analysis

The continuous data in normal distribution will be expressed as mean ± standard deviation (mean ± SD), and differences between two groups are compared using *t* test, paired *t* test, or analysis of variance (ANOVA). The continuous data in skewed distribution will be expressed as medians and interquartile ranges (IQRs), and differences between the two groups are compared using a rank-sum test. The categorical data will be expressed as number (*n*) and percentage (%), and differences between the two groups are compared using the *χ*^2^ test or Fisher’s exact test. For comprehensive efficacy analysis of multi-centers, categorical data will be compared using the Cochran-Mantel-Haenszel *χ*^2^ test, and continuous data will be compared using analysis of covariance. SAS 9.4 (SAS Institute Inc., Cary, NC, USA) will be used for statistical analysis, and *P* < 0.05 is regarded as the statistical significance.

## Discussion

FAP is a nonorganic pain, which seriously affects the mental health and life quality of patients [4]. Previous studies have initially investigated the efficacy of acupuncture on FAP [10, 11]. Cheng et al. have reported that electroacupuncture at Baliao acupoint is effective in the decreased pain level and improved quality of life, while their study is performed in a single center [10]. Yu et al. have also reported the good clinical efficacy of electroacupuncture at Baliao acupoint in FAP, while the sample size of their study is limited, with 48 cases in total [11]. Therefore, this protocol designs a multi-center (4 centers), randomized controlled trial with a larger sample size (136 cases) to further explore the efficacy of acupuncture on FAP.

Acupuncture has been applied for more than 3000 years in China and is commonly used to relieve pain, such as postoperative pain, musculoskeletal pain, and migraine prophylaxis [20–23]. Acupuncture may achieve pain relief by improving microcirculation to eliminate pathological factors that cause pain or by blocking the continuous circulation of pain sensation [24]. In terms of the occurrence of pain, FAP may occur due to abnormal changes in blood flow, nerves, and muscles [25]. Baliao acupoint is a traditional Chinese acupuncture point and is divided into four pairs: Shangliao (BL31), Ciliao (BL32), Zhongliao (BL33), and Xialiao (BL34), with a total of 8 acupuncture points [8]. Deeply needling the Baliao acupoint, the needle sensation transmitted to the anus highly overlaps with the signals outgone by efferent nerve of pelvic organs, and perianal blood circulation is promoted through neurohumoral regulation to improve anal muscle imbalance, reduce anal pressure, and restore anal function [26]. In electroacupuncture therapy, dense wave can sequentially inhibit sensory neurons and motor neurons, reduce nerve stress function, and alleviate muscle tissue spasms to achieve instant analgesia [11]. Sparse wave can cause muscle contraction, generate strong tremors, dilate local blood vessels, and improve material exchange efficiency between tissues and blood to promote self-repair of damaged neuromuscular tissues [11]. The alternating effect of sparse wave and dense wave can provide a quick analgesic effect while repairing the unstable state of pelvic floor tissues and organs [11].

There are several strengths in this trial. First, the sample size of this trial is bigger than previous studies. Second, this study is carried out in multi-centers, which makes the results more reliable. Also, there are some limitations in this trial. First, it is difficult to make operators and patients be blind due to the particularity of acupuncture operation. Although shallow needling will be performed in the control group, the bias may exist. Second, the acupuncture will be performed by different acupuncturists, which may cause potential bias. Third, the applicability of this acupuncture in patients with a history of anal and pelvic floor surgery is unclear because this population is excluded.

This trial enhances the stimulation of acupuncture on Baliao acupoint through electroacupuncture, which will help to guide decisions about the treatment of FAP patients and promote the clinical application of electroacupuncture in analgesia. However, there are some limitations in this trial.

## Supplementary Information

Below is the link to the electronic supplementary material.

Supplementary file1 (DOCX 28 KB)

## Data Availability

The datasets used and/or analyzed during the current study are available from the corresponding author on reasonable request.

## References

[1] Rongqing G, Yafei W, Zhimin W, Feng L, Yuantao L, Xinhua C, Lu C, Hui Z, Kailun L (2019) Treatment outcome of acute sacral nerve stimulation in functional anorectal pain. Pain Pract 19(4):390–396. 10.1111/papr.12751 30472789 10.1111/papr.12751

[2] Drossman DA, Hasler WL (2016) Rome IV-functional GI disorders: disorders of gut-brain interaction. Gastroenterology 150(6):1257–1261. 10.1053/j.gastro.2016.03.035 27147121 10.1053/j.gastro.2016.03.035

[3] Rao SS, Bharucha AE, Chiarioni G, Felt-Bersma R, Knowles C, Malcolm A, Wald A (2016) Functional anorectal disorders. Gastroenterology. 10.1053/j.gastro.2016.02.009 27144630 10.1053/j.gastro.2016.02.009 PMC5035713

[4] Zhang Q, Liu Y, Zhang Q, Zhang Y, Wu S, Jiang B, Ni M (2020) Impaired anorectal afferents is a potential pathophysiological factor associated to functional anorectal pain. Front Neurol 11:577025. 10.3389/fneur.2020.577025 33162929 10.3389/fneur.2020.577025 PMC7581696

[5] Chiarioni G, Nardo A, Vantini I, Romito A, Whitehead WE (2010) Biofeedback is superior to electrogalvanic stimulation and massage for treatment of levator ani syndrome. Gastroenterology 138(4):1321–1329. 10.1053/j.gastro.2009.12.040 20044997 10.1053/j.gastro.2009.12.040 PMC2847007

[6] Lascano AM, Lalive PH, Hardmeier M, Fuhr P, Seeck M (2017) Clinical evoked potentials in neurology: a review of techniques and indications. J Neurol Neurosurg Psychiatry 88(8):688–696. 10.1136/jnnp-2016-314791 28235778 10.1136/jnnp-2016-314791

[7] Byrnes KG, Sahebally SM, McCawley N, Burke JP (2022) Optimal management of functional anorectal pain: a systematic review and network meta-analysis. Eur J Gastroenterol Hepatol 34(3):249–259. 10.1097/meg.0000000000002222 34091479 10.1097/MEG.0000000000002222

[8] Shen J, Zhou X, Zhao J, Wang H, Ye T, Chen W, Wang X, Gong L, Cai Y (2023) Electroacupuncture at Baliao point alleviates post-operative pain and anal distension after procedure for prolapse and hemorrhoids (stapled hemorrhoidopexy): a prospective randomized clinical trial. Int J Colorectal Dis 38(1):104. 10.1007/s00384-023-04403-y 37074488 10.1007/s00384-023-04403-y PMC10115677

[9] Liu Y, Liu L, Wang X (2013) Electroacupuncture at points Baliao and Huiyang (BL35) for post-stroke detrusor overactivity. Neural Regen Res 8(18):1663–1672. 10.3969/j.issn.1673-5374.2013.18.004 25206463 10.3969/j.issn.1673-5374.2013.18.004 PMC4145909

[10] Cheng S, Gao H, Xiong G, Zhang S, Sha X, Min L, Ying G (2021) Clinical observation on the therapeutic effect of electroacupuncture at Baliao acupoint on chronic anal pain. World Journal of Integrated Traditional and Western Medicine 16(5):961–963

[11] Yu Y, Yin L, Yao Q, Chen M, Yang W, Pei C (2016) Evaluation of therapeutic effect of electroacupuncture combined with biofeedback on functional anorectal pain. J Tradit Chin Med 23(6):696–697

[12] Moher D, Schulz KF, Altman DG (2001) The CONSORT statement: revised recommendations for improving the quality of reports of parallel group randomized trials. BMC Med Res Methodol 1:2. 10.1186/1471-2288-1-2 11336663 10.1186/1471-2288-1-2 PMC32201

[13] MacPherson H, White A, Cummings M, Jobst K, Rose K, Niemtzow R (2001) Standards for reporting interventions in controlled trials of acupuncture: the STRICTA recommendations. Complement Ther Med 9(4):246–249. 10.1054/ctim.2001.0488 12184354 10.1054/ctim.2001.0488

[14] Zheng X (2002) Guiding rules of clinical research of new Chinese medicine. Chinese Medicine Publishing House

[15] Ware JE Jr, Sherbourne CD (1992) The MOS 36-item short-form health survey (SF-36). I. Conceptual framework and item selection. Med Care 30(6):473–483 1593914

[16] Zung WW (1971) A rating instrument for anxiety disorders. Psychosomatics 12(6):371–379. 10.1016/s0033-3182(71)71479-0 5172928 10.1016/S0033-3182(71)71479-0

[17] Zung WW, Richards CB, Short MJ (1965) Self-rating depression scale in an outpatient clinic. Further validation of the SDS. Arch Gen Psychiatry 13(6):508–515. 10.1001/archpsyc.1965.01730060026004 4378854 10.1001/archpsyc.1965.01730060026004

[18] Detsomboonrat P, Pisarnturakit PP (2019) Development and evaluation: the satisfaction of using an oral health survey mobile application. Telemed J E Health 25(1):55–59 29870315 10.1089/tmj.2017.0288

[19] Tu JF, Cao Y, Wang LQ, Shi GX, Jia LC, Liu BL, Yao WH, Pei XL, Cao Y, Li HW, Yan SY, Yang JW, Qu ZC, Liu CZ (2022) Effect of adjunctive acupuncture on pain relief among emergency department patients with acute renal colic due to urolithiasis: a randomized clinical trial. JAMA Netw Open 5(8):e2225735. 10.1001/jamanetworkopen.2022.25735 35943743 10.1001/jamanetworkopen.2022.25735 PMC9364130

[20] Liu L, Yuan X, Yang L, Zhang J, Luo J, Huang G, Huo J (2020) Effect of acupuncture on hormone level in patients with gastrointestinal dysfunction after general anesthesia: a study protocol for a randomized controlled trial. Medicine 99(14):e19610. 10.1097/md.0000000000019610 32243385 10.1097/MD.0000000000019610 PMC7440186

[21] Shah S, Godhardt L, Spofford C (2022) Acupuncture and postoperative pain reduction. Curr Pain Headache Rep 26(6):453–458. 10.1007/s11916-022-01048-4 35482244 10.1007/s11916-022-01048-4

[22] Yuan QL, Wang P, Liu L, Sun F, Cai YS, Wu WT, Ye ML, Ma JT, Xu BB (2016) Zhang YG (2016) Acupuncture for musculoskeletal pain: a meta-analysis and meta-regression of sham-controlled randomized clinical trials. Sci Rep 6:30675. 10.1038/srep30675 27471137 10.1038/srep30675 PMC4965798

[23] Zhao L, Chen J, Li Y, Sun X, Chang X, Zheng H, Gong B, Huang Y, Yang M, Wu X, Li X, Liang F (2017) The long-term effect of acupuncture for migraine prophylaxis: a randomized clinical trial. JAMA Intern Med 177(4):508–515. 10.1001/jamainternmed.2016.9378 28241154 10.1001/jamainternmed.2016.9378

[24] Wu MS, Chen KH, Chen IF, Huang SK, Tzeng PC, Yeh ML, Lee FP, Lin JG, Chen C (2016) The efficacy of acupuncture in post-operative pain management: a systematic review and meta-analysis. PLoS ONE 11(3):e0150367. 10.1371/journal.pone.0150367 26959661 10.1371/journal.pone.0150367 PMC4784927

[25] Bharucha AE, Wald A, Enck P, Rao S (2006) Functional anorectal disorders. Gastroenterology 130(5):1510–1518. 10.1053/j.gastro.2005.11.064 16678564 10.1053/j.gastro.2005.11.064

[26] Song Y, Ni G (2019) Electroacupuncture at Baliao point for the treatment of severe anal prolapse and distension in patients undergoing mixed hemorrhoid surgery. Chinese Acupuncture and Moxibustion 39(9):970 10.13703/j.0255-2930.2019.03.008 30942010

