# Supplementary material for: Efficacy of electroacupuncture therapy in patients with functional anorectal pain: study protocol for a multicenter randomized controlled trial
Source: Int J Colorectal Dis. 2024 Apr 22;39(1):55. doi: 10.1007/s00384-024-04628-5 (PMC11035392; doi:10.1007/s00384-024-04628-5)
Supplement: Supplementary file 1 — Supplementary file1 (DOCX 28 KB) [file 384_2024_4628_MOESM1_ESM.docx]

**Supplementary Table 1 Schedule of this trial**

|  | Study period | | | | | | | | | | | | |
| --- | --- | --- | --- | --- | --- | --- | --- | --- | --- | --- | --- | --- | --- |
|  | Baseline | Allocation | Treatment | | | | | | | | Follow-up | | |
| Timepoints (weeks) | -2 | 0 | 1 | 2 | 3 | 4 | 5 | 6 | 7 | 8 | 12 | 20 | 32 |
| Recruitment |  |  |  |  |  |  |  |  |  |  |  |  |  |
| Eligibility assessment | X |  |  |  |  |  |  |  |  |  |  |  |  |
| Informed consent | X |  |  |  |  |  |  |  |  |  |  |  |  |
| Demographic data | X |  |  |  |  |  |  |  |  |  |  |  |  |
| Allocation |  | X |  |  |  |  |  |  |  |  |  |  |  |
| Intervention |  |  |  |  |  |  |  |  |  |  |  |  |  |
| Electroacupuncture group |  |  |  |  |  |  |  |  |  |  |  |  |  |
| Sham electroacupuncture group |  |  |  |  |  |  |  |  |  |  |  |  |  |
| Outcome measurement |  |  |  |  |  |  |  |  |  |  |  |  |  |
| Clinical treatment efficacy |  |  |  |  |  | X |  |  |  | X | X | X | X |
| VAS score |  | X |  |  |  | X |  |  |  | X | X | X | X |
| Pain days per month |  | X |  |  |  | X |  |  |  | X | X | X | X |
| Quality of life |  | X |  |  |  | X |  |  |  | X | X | X | X |
| Psychological state assessment |  | X |  |  |  |  |  |  |  | X |  | X | X |
| Anorectal manometry |  | X |  |  |  |  |  |  |  | X |  |  | X |
| Pelvic floor electromyography |  | X |  |  |  |  |  |  |  | X |  |  | X |
| Patient satisfaction |  |  |  |  |  |  |  |  |  |  |  |  | X |

VAS, visual analogue scale.
